# Changing education landscapes and (in)equality in Wales: An exploration of barriers to tertiary education using linked UK Census and education data

**DOI:** 10.23889/ijpds.v9i5.2500

**Published:** 2024-09-18

**Authors:** Katy Huxley, Rhys Davies

**Affiliations:** 1 Cardiff University

## Objective

The Wales Commission for Tertiary Education and Research (CTER) was created in 2024 to oversee a complex system of training and education for adults and young people. The objective of our research was to examine demographic features that prevent or encourage engagement with education after formal schooling, that can be used to inform future CTER strategies.

## Approach

This research considered the personal and family characteristics of learners, and evaluated differentials between characteristics in terms of enrolment, progression/exit and completion in different tertiary education settings (Sixth Forms, Further Education, and Higher Education). Using novel data linkages across 5 different data sources, we used household characteristics from the UK Census 2011 to help contextualise the experiences of learners and explore the manifestation of inequalities in the tertiary education system.

## Results

The results indicated that inequities were apparent. Gender, Special Education Needs (SEN) status, and household characteristics including disability, deprivation, socio-economic, occupational and education status’ created barriers to continued engagement in education post schooling.

## Conclusions and Implications

The complex and disparate nature of data collections was highlighted by the research, whilst the findings offer areas and characteristics that CTER should consider when creating new initiatives or governance systems for the tertiary education sector in Wales in order to reduce persistent inequalities. Recommendations for CTER that were drawn from the research included: 1) a more cohesive approach to data collection across the tertiary sector, and 2) strategies should assess impacts for those experiencing multiple forms of disadvantage.

